# The Role of Treatment Sequencing with Immune-Checkpoint Inhibitors and BRAF/MEK Inhibitors for Response and Survival of Patients with BRAFV600-Mutant Metastatic Melanoma—A Retrospective, Real-World Cohort Study

**DOI:** 10.3390/cancers14092082

**Published:** 2022-04-21

**Authors:** Maximilian Haist, Henner Stege, Ronja Ebner, Maria Isabel Fleischer, Carmen Loquai, Stephan Grabbe

**Affiliations:** Department of Dermatology, University Medical Center Mainz, 55131 Mainz, Germany; henner.stege@unimedizin-mainz.de (H.S.); ronja.25@web.de (R.E.); mariaisabel.fleischer@unimedizin-mainz.de (M.I.F.); carmen.loquai@unimedizin-mainz.de (C.L.); stephan.grabbe@unimedizin-mainz.de (S.G.)

**Keywords:** metastatic melanoma, sequential treatment, immune-checkpoint inhibitors, BRAF/MEK-inhibitors, targeted therapy, BRAF-mutation

## Abstract

**Simple Summary:**

The clinical application of kinase inhibitors and immune-checkpoint inhibitors (CPI) has substantially improved the treatment landscape of melanoma. While BRAF/MEK inhibitors allow for rapid disease control, and CPI can evoke durable tumor responses, primary and secondary resistance frequently limit treatment efficacy. Observations from preclinical trials suggest that treatment with BRAF/MEK inhibitors may enhance susceptibility towards CPI therapy and thus improve long-term tumor control. To date, little prospective data is available for the optimal sequencing of these agents in melanoma patients. In this retrospective, real-world analysis, we analyzed the role of sequential treatment with BRAF/MEK inhibitors and CPI in 135 BRAF-mutant, metastatic melanoma patients. Our results demonstrate that front-line treatment with CPI is associated with favorable tumor control and overall survival in patients with BRAF-mutant melanoma. Further, our data indicate that patients who are refractory to front-line BRAF/MEKi therapy are at higher risk of rapid disease progression compared to patients with front-line CPI treatment.

**Abstract:**

The advent of immune-checkpoint inhibitors (CPI) and BRAF/MEK-directed targeted therapy (TT) has improved the treatment landscape of patients with BRAFV600-mutant metastatic melanoma. While TT allows for rapid disease control, the development of secondary TT resistance limits the duration of responses. Responses to CPI have a slower onset but can be durable in a subset of patients. To date, little prospective data is available for the optimal sequencing of these agents in melanoma patients. In this retrospective, single-center, real-world analysis, we identified 135 patients with BRAF-mutated, metastatic melanoma who received consecutive treatment with TT followed by CPI, or vice versa, as first and second-line therapy, respectively. We collected data on clinical-pathological factors, treatment duration, best overall response, progression-free survival and overall survival (OS). Our data revealed that front-line treatment with CPI, followed by TT, showed a non-significant trend towards better OS compared to front-line TT (median OS: 35.0 vs. 18.0 months, *p* = 0.070). This association was confirmed in a subgroup of patients without systemic pre-treatments (median OS: 41.0 vs. 14.0 months, *p* = 0.02). Further, we observed significantly better objective response rates to second-line treatments for patients receiving front-line CPI (18.4 vs. 37.8%, *p* = 0.024). Last, our results indicated that rapid disease progression was less common in patients treated with front-line CPI (27.6% vs. 16.2%) and that subsequent treatment with TT resulted in favorable survival outcomes. Our real-world data indicate that sequential treatment with front-line CPI is associated with favorable tumor control and overall survival in a subgroup of previously untreated BRAF-mutant metastatic melanoma patients.

## 1. Introduction

Within the last decades, the incidence of malignant melanoma has dramatically increased and is responsible for over 90% of skin cancer deaths in the western world [1]. In parallel, the understanding of the underlying molecular biology of melanoma has resulted in significant advances in the treatment of metastatic melanoma, and new systemic treatment options have emerged in recent years. In this regard, the approval of checkpoint inhibitors (CPI) and BRAF/MEK-directed targeted therapies (TT) has significantly improved the survival of melanoma patients with a BRAF-V600 mutation [2,3]. 

About half of all melanoma patients harbor an activating BRAF mutation that results in the constitutive activation of the RAF-MEK-ERK signal transduction pathway, which is critical for melanoma development and progression [4]. Therapeutic targeting of this pathway has significantly prolonged overall (OS) and progression-free survival (PFS) in the last decade, and even durable tumor responses have been observed. In particular, it has been demonstrated that first-line BRAF/MEK-inhibitor (BRAF/MEKi) therapy evoked complete responses (CR) in 11–19% of BRAF-mutant metastatic melanoma patients with a median PFS between 11.1–14.9 months [5,6,7]. At the same time, five-year survival rates reached 34% for dabrafenib + trametinib, demonstrating sustained tumor responses [5].

In addition, patients with a BRAF mutation are also eligible for CPI treatment, which demonstrated impressive improvements in survival data. Specifically, in the CheckMate067 trial, 22% of treatment-naïve metastatic melanoma patients achieved a CR upon combined checkpoint-inhibitor therapy with ipilimumab + nivolumab with slightly better survival outcomes for BRAF-mutant melanoma patients, which showed a median PFS of 16.8 months [8]. For this subgroup of patients, five-year survival rates were 60%, compared to 46% and 30% for nivolumab or ipilimumab monotherapy, respectively [8].

Regardless of these promising survival data, recent evidence shows that approximately 70–80% of patients with metastatic melanoma harboring a BRAF-mutation and treated with CPI or TT will, at some time, show a relapse of the disease in a real-world setting, which requires the re-initiation of anti-tumor treatments [9]. Furthermore, due to the lack of head-to-head trials comparing the different treatment regimens, it remains an unsolved issue for dermato-oncologists to determine (a) the most suitable first-line therapeutic option, and (b) the optimal treatment sequencing of BRAF/MEKi therapy and CPI, which might be particularly relevant in relation to the observation that cross-resistance of TT and CPI exists at a molecular level [10].

In routine clinical practice, BRAF/MEK-directed targeted therapy is regularly applied as a frontline therapy of choice in patients with advanced and aggressively growing metastatic melanoma who require immediate disease control, due to its rapid onset of activity and high response rates, even in patients with high lactate dehydrogenase (LDH) levels [11]. While TT allows for rapid disease control in patients with a high tumor burden, high LDH levels and symptomatic disease, the duration of response is often limited in these patients, and disease progression due to secondary acquired resistance frequently occurs after 13–15 months [12,13]. While acquired resistance is the major challenge for TT, primary refractory disease is more common for patients receiving CPI [8]. Treatment with CPI is characterized by a lower rate of objective responses and a slower onset of action, while long-term follow-ups from clinical trials have demonstrated that sustained and profound responses occur in a subset of patients and may even continue after treatment discontinuation [14]. However, the time until the onset of response of CPI is variable and patients with multifocal metastatic disease and elevated LDH levels are less likely to respond [15]. Therefore, front-line CPI is often favored in asymptomatic patients with low tumor burden and normal LDH levels, although combined CPI therapy has certainly also shown a rapid onset of action in patients with a high tumor burden [16].

To improve patient outcomes, there are currently several prospective trials that are investigating strategies with sequential TT and CPI (see Supplementary Table S6). Supporting sequential treatment strategies are preclinical studies that suggest that TT might enhance the susceptibility to CPI by modulating the tumor microenvironment (TME), thus indicating that TT and CPI may act synergistically [17,18]. In particular, it has been shown that vemurafenib may increase antigen presentation by melanoma cells and their recognition by melanoma-specific T-cells [19]. Hence, it has been hypothesized that pre-treatment with TT might improve CPI efficacy in a clinical setting by decreasing tumor burden and normalizing LDH, while avoiding the culminating toxicity of a triple therapy regimen of CPI + TT [20]. However, these preclinical observations have not been confirmed in retrospective studies, which found that patients with front-line BRAF/MEKi therapy showed worse survival data [21,22]. While this observation might be inferred from worse prognostic features found in patients treated with front-line TT, it has also been reasoned that these patients might not receive optimal clinical benefit from subsequent CPI due to insufficient time to complete enough cycles of immunotherapy. In this regard, Johnson and coworkers further showed that TT after anti-PD1 therapy is less effective, thus challenging the dogma that TT might be equally effective, irrespective of being administered before or after CPI therapy, suggesting a shared responder phenotype [23].

With little data available so far from prospective, head-to-head comparisons of therapy sequencing in BRAF-mutated melanoma patients, and conflicting data from previous clinical trials on this issue, it remains unclear which treatment sequence might be best for patients with BRAF-mutant metastatic melanoma. In this single-center retrospective analysis, we aimed to determine if the sequence in which BRAF/MEKi and CPI are administered affected the clinical outcome in our real-world patient cohort. In particular, we hypothesized that sequential treatment with front-line BRAF/MEKi therapy, followed by CPI treatment, would be associated with better response and survival than the vice versa approach. Further, we aimed to identify factors that could potentially be used to guide treatment-sequencing decisions.

## 2. Patients and Methods

### 2.1. Patient Population

**Figure 1:**
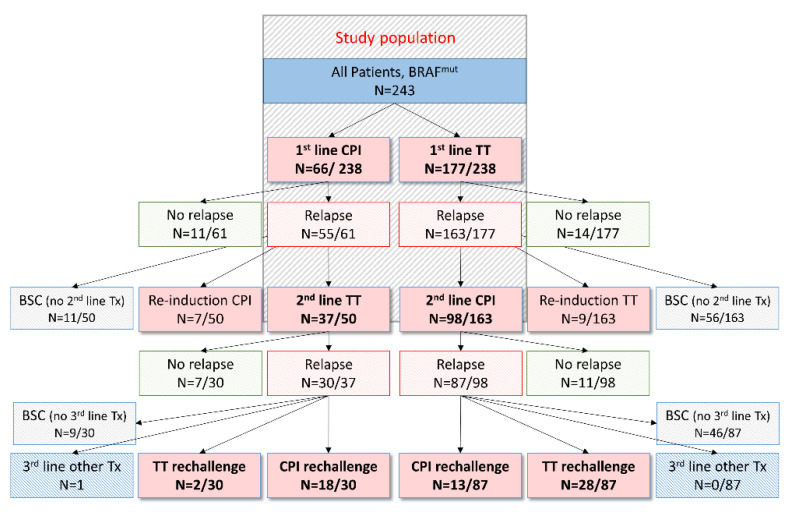
Flow chart showing the selection steps of the retrospective study and the treatment outcomes of melanoma patients after front-line treatment. We included all patients with stage IV melanoma who had confirmed BRAF-V600 mutation. Among the 243 patients, 66 patients received front-line (1L) CPI therapy and 177 were treated with front-line (1L) BRAF ± MEKi therapy. Following tumor progression upon 1L BRAF ± MEKi therapy, 98 patients subsequently received CPI in a second-line (2L) setting. Among these 98 patients, 87 showed tumor progression, comprising 27 rapid progressors, that did not allow for the initiation of another treatment line in 11 patients. Among the remaining 60 patients, 41 patients were again treated with CPI, BRAF ± MEKi therapy or chemotherapy. On the other hand, patients showing disease progression upon 1L CPI therapy (*n =* 55), were either rechallenged with CPI (*n =* 7) or received BRAF ± MEKi therapy in a 2L setting (*n =* 37). Among these patients, 30 again showed tumor progression, which resulted in the initiation of 3L treatment in 21 patients. Abbreviations: BSC = best supportive care; CPI = immune-checkpoint-inhibitor therapy; TT = targeted therapy; Tx = treatment.

In this single-institution retrospective analysis, we report on the outcomes of patients with BRAF-mutant metastatic melanoma who received sequential treatment with BRAF ± MEKi and CPI, or vice versa, at the University Medical Center, Mainz, between March 2011 and March 2021, with follow-up until October 2021. Patients were eligible for analysis if they had histologically confirmed stage IV, BRAF-V600 mutant melanoma, and received consecutive treatment with BRAF ± MEKi (such as monotherapy with the BRAFi vemurafenib, dabrafenib or encorafenib, or the MEKi cobimetinib, trametinib or binimetinib, or the corresponding combinations of BRAF + MEKi) and CPI (such as combination immunotherapy with ipilimumab + nivolumab, or monotherapy with ipilimumab, nivolumab or pembrolizumab), or vice versa, for at least one month. Patients who had received BRAF ± MEKi or CPI therapy in an adjuvant setting were excluded from further analysis. In total, we identified 135 patients (72 male and 63 female) who received front-line BRAF ± MEKi therapy (first-line; 1L) followed by CPI (second-line; 2L), and vice versa. These were recruited from a larger cohort of 243 metastatic melanoma patients with proven BRAF-V600 mutation (see Figure 1), some of whom (*n =* 95) were included from a previously published retrospective analysis of advanced melanoma patients who had been treated with BRAF ± MEKi and/or CPI at the University Medical Center, Mainz [24]. The data cut-off was set at October 2021.

Data on baseline demographics, tumor specifics (i.e., BRAF-status, tumor thickness, ulceration, AJCC stage at treatment initiation, and localization of metastases), laboratory results, primary and secondary clinical outcomes, as well as data on previous systemic treatments (mainly in an adjuvant setting), front-line and second-line treatments (i.e., treatment regimen, treatment duration, tumor progression, cessation due to adverse events, AEs), time-to-next-treatment (TTNT), treatment regimens after 2L treatment, and the status of the patient at the time of data lock (October 2021) were collected by electronic chart review. Clinical decisions regarding the prescription of BRAF ± MEKi and CPI were made independently of this study. BRAF mutations were mainly assessed by the Department of Pathology of the University Medical Center, Mainz, using next-generation sequencing with the nNGM plus Booster Panel (Qiagen, Hilden, Germany) and the MiSeq Illumina sequencing platform, with data analysis being conducted with the CLC Genomics Workbench (Qiagen). 

### 2.2. Primary and Secondary Clinical Outcomes

We analyzed the impact of treatment sequencing with BRAF ± MEKi and CPI on the clinical outcomes of patients. We divided patients into two cohorts: those who had received CPI before TT (CPI first) and those who received TT before CPI (TT first). Due to the different mode of actions of BRAF ± MEKi and the different response profiles, we defined OS as the primary clinical outcome in this analysis, which was assessed from the initiation of 1L treatment. Secondary clinical outcomes included the best overall response (BOR) to 1L and 2L therapy, objective response rate (ORR), disease control rate (DCR), and PFS upon initiation of 1L and 2L treatment (for details see Supplementary Table S1). BOR was defined as complete response (CR), partial response (PR), stable disease (SD), or progressive disease (PD) according to the Revised Response Evaluation Criteria in Solid Tumors (RECIST) 1.1. guidelines. ORR was defined as the proportion of patients with CR or PR, and DCR was the proportion of patients with CR, PR or SD.

### 2.3. Statistical Analysis

Descriptive statistics were used to analyze the baseline characteristics of the study population. Treatment duration was calculated as the period between initial drug administration and treatment discontinuation. PFS was calculated from the start of front-line treatment (BRAF ± MEKi or CPI therapy) to the date of radiological or clinical disease progression, last follow-up, or death from any cause. OS was calculated from the start of front-line treatment to the date of death or last follow-up, unless described otherwise. The Chi-square test was used to assess the association between the different treatment sequences and clinicopathological features. The Clopper–Pearson method was used to calculate 95% confidence intervals (CI) for the categorical variables. Testing for equality between patients receiving front-line CPI followed by BRAF ± MEKi, or vice versa, was performed using Student’s *t*-test, Mann-Whitney test (in case of non-parametric comparisons) or Chi-square test. Comparisons between continuous variables of the different treatment sequences were performed using ANOVA variance analysis. 

We employed Kaplan–Meier survival plots to illustrate median OS and PFS probabilities and to explore the association between the different treatment sequences, PFS and OS. Survival curves were compared using log-rank tests. The median duration of follow-up was calculated using the reverse Kaplan–Meier method. Cox’s proportional hazards models were applied to identify the strongest predictors for survival analyses by adjusting for baseline characteristics, treatment sequence, and laboratory results. Here, hazards ratios (HR) were provided with 95% confidence intervals (CI). Multivariate analysis was calculated for the significant (*p* ≤ 0.05) variables by the univariate test or a priori selection for biological relevance to evaluate their conjoint, independent effects on OS. In all cases, two-tailed *p*-values were calculated and considered significant with value of *p* < 0.05. SPSS (version 27, IBM, Ehningen, Germany), RStudio (Version 1.3.1093), and GraphPad PRISM (Version 5, San Diego, CA, USA) were used for all analyses.

## 3. Results

### 3.1. Patients’ Characteristics

A total of 135 patients (72 male and 63 female) who received first-line BRAF ± MEKi (1L) and subsequent treatment with CPI (2L), or vice versa, were included in the retrospective analysis on the impact of treatment sequencing for patient survival. These patients were selected from a cohort of 243 metastatic melanoma patients who tested positive for the BRAF-V600 mutation. Some of the patients were recruited from a previously published retrospective analysis of advanced melanoma patients treated with BRAF ± MEKi and/or CPI between 2011 to 2021 [24]. The patient cohort was biased towards more advanced disease as the majority of the patients presented with adverse prognostic factors at initiation of 1L treatment. 

Among the 243 patients showing a BRAF-V600 mutation, 66 received 1L treatment with CPI, and 177 first received BRAF ± MEKi. Most of these patients experienced tumor progression in the follow-up period from March 2011 until October 2021. In particular, 16.7% of patients with 1L CPI therapy showed an ongoing response upon 1L CPI therapy, whereas only 7.9% of patients treated with 1L BRAF ± MEKi did not experience tumor progression at any time during the follow-up period (see Figure 1). In most of the 218 BRAF-mutant melanoma patients showing tumor progression, 2L treatment was initiated, of which 135 patients received BRAF ± MEKi and CPI in sequence (55.6%) and were thus eligible for our analysis. These 135 patients comprised 98 patients (72.6%) who received CPI upon disease progression with BRAF ± MEKi and 37 patients (27.4%) who received BRAF ± MEKi upon disease progression with CPI. 

The median age at initiation of 1L treatment was 59.0 years. All 135 patients in this cohort were diagnosed with BRAF-mutant melanoma (13 patients tested positive for BRAF-V600K mutation, 105 tested positive for BRAF-V600E mutation, and in 17 patients PCR test was unable to distinguish between either BRAF-V600E/K mutation) and some patients showed an additional mutation, such as NRAS p.Q61 (*n =* 6) or EGFR (*n =* 1). The median Breslow thickness was 2.3 mm, and 54.8% of melanomas were ulcerated at primary diagnosis. Baseline LDH serum levels were elevated in 68 patients (72.3%) and 33 patients presented with LDH-levels elevated by >1.5-fold (35.1%). In the course of the disease, 69 patients (51.1%) developed melanoma brain metastasis (MBM) and 49 patients (36.3%) presented with hepatic metastasis at the time of initiation of 1L treatment. In addition, 42 patients received at least one line of systemic therapy prior to the initiation of either BRAF ± MEKi or CPI therapy (31.1%). Systemic pretreatments comprised IFN-α-treatment (34/42), CPI, targeted therapy or study medications in an adjuvant setting. The median time to initiation of BRAF ± MEKi or CPI upon prior adjuvant treatment was 15.0 months. Notably, all patients showed distant metastasis upon initial application of BRAF ± MEKi or CPI. 

Among the 135 patients who received CPI followed by BRAF ± MEKi therapy, 17 (12.6%) initially received combined CPI therapy, whereas 20 (14.8%) were given CPI monotherapy, which comprised of nivolumab or pembrolizumab (*n =* 14), or ipilimumab (*n =* 6). The median duration of the initial CPI treatment was 3.0 months, with 16 patients (43.2%) ceasing CPI therapy due to the occurrence of AEs, which was significantly more often compared to the cohort receiving front-line BRAF ± MEKi therapy (*n =* 6; 6.1%; *p* < 0.001). On the other hand, 60 patients (44.4%) were initially treated with a combination of BRAF + MEK-inhibitors and 38 patients only received BRAF-inhibitor monotherapy (28.1%, patients were treated before combined BRAF/MEKi therapy became widely available). Front-line BRAF ± MEKi therapy was given for a median treatment duration of 6.0 months. The number of systemic pre-treatments was similar in both arms. 

As an inclusion criterion for our analysis, all 135 patients had shown tumor progression upon initial CPI or BRAF ± MEKi treatment, which required the administration of subsequent tumor treatments. The median time until the introduction of the subsequent treatment line was 1.0 month. Notably, the median TTNT was longer for patients being initially treated with CPI compared to patients receiving BRAF ± MEKi in a first-line setting (3.0 months vs. 1.0 months; *p* = 0.102). The 2L treatments comprised either combined CPI therapy (*n =* 41), anti-PD1 monotherapy (*n =* 29), ipilimumab (*n =* 28), combined BRAF/MEKi therapy (*n =* 29) or BRAFi or MEKi monotherapy (*n =* 8). The median duration of this 2L therapy was 3.0 months. In the course of this 2L treatment 116/135 patients (85.4%) developed tumor progression, which required the initiation of subsequent treatment lines. By contrast, 19 patients receiving 2L treatments did not show tumor progression during the overall observation period and 9 patients still received 2L treatments at the time of data-lock including 5 patients initially treated with BRAF ± MEKi therapy and 4 patients with initial CPI therapy (*p* = 0.257). 

Patients requiring further treatment upon tumor progression with 2L treatment received a median of 1.5 subsequent treatment lines (range: 1–3), which comprised either a rechallenge with BRAF ± MEKi (*n =* 47), CPI-rechallenge (*n =* 50) or other treatments (i.e., study medications or chemotherapy; *n =* 3). The median time until the initiation of subsequent treatment lines was 2.0 months. During the overall observation period, 68 patients died (50.4%) with a median OS of 33.0 months (95% CI: 22.1–43.9 months). Median follow-up time of the study cohort from the start of first systemic treatment was 58.0 months (95% CI: 42.4–73.6 months) and median follow-up from initiation of 1L treatment was 41.0 months (95% CI: 31.6–50.4 months). 

Further details on baseline patient characteristics and the subgroups stratified by the therapeutic sequence of BRAF ± MEKi and CPI are given in Table 1. No significant differences in terms of clinical and biological characteristics were observed between patients first given BRAF ± MEKi followed by CPI therapy (group 1) vs. patients starting with CPI therapy and subsequent BRAF ± MEKi therapy (group 2), although there was a trend that patients with front-line BRAF ± MEKi therapy more often presented with thicker and ulcerated (*p* = 0.032) melanomas. Further, it was found that patients given BRAF ± MEKi received front-line treatment for a significantly longer time period than patients first given CPI (5.0 vs. 3.0 months; *p* = 0.022). Patients initially treated with BRAF ± MEK-inhibitors had a shorter TTNT upon tumor progression (1.0 vs. 3.0 months; *p* = 0.09). On the other hand, patients receiving BRAF ± MEKi after initial CPI therapy were receiving 2L treatment with BRAF ± MEKi for a significantly longer time period compared to patients receiving CPI after initial BRAF ± MEK application (4.0 vs. 3.0 months; *p* = 0.009). We observed a trend, that patients given BRAF/MEKi after initial CPI, more often received combined BRAF + MEKi therapy compared to patients first treated with TT, albeit this association was below statistical significance (two-tailed t-test; *p* = 0.062). Of note, the median follow-up time for both patient cohorts calculated from the initiation of 1L treatment was not significantly different (43.0 months vs. 41.0 months, *p* = 0.159). 

### 3.2. Factors Associated with Disease Progression and Survival

To allow for a comparison of the investigated cohort with previously described cohorts of metastatic melanoma patients treated with different sequencing regimens of BRAF/MEK inhibitors and CPI, we used Cox regression analysis to identify clinical and biological factors affecting survival upon melanoma treatment.

The best predictors of survival in univariate analysis in this patient cohort with tumor progression upon 1L treatment were elevated serum LDH-levels, the presence of melanoma brain metastases (MBM), the presence of hepatic metastasis, the objective response rate to 1L or 2L therapy and the duration of 1L and 2L treatment (Supplementary Table S2). Given the number of target events and the biological rationale, we included the statistically significant factors into a multivariate analysis. This multivariate model confirmed an independent association of the BOR with 2L treatment (HR: 0.34, 95% CI: 0.13–0.92, *p* = 0.02), the duration of 2L treatment (HR: 0.23, 95% CI: 0.10–0.53, *p* = 0.001), the presence of liver metastasis (HR: 2.12, 95% CI: 1.03–4.38, *p* = 0.04) and LDH-serum levels at baseline (HR: 2.26, 95% CI: 1.02–4.98, *p*= 0.044) with the overall survival (Supplementary Table S3). Notably, the applied treatment sequence (front-line CPI vs. front-line BRAF/MEKi therapy) was not significantly associated with overall survival in this multivariate Cox-regression analysis (HR: 0.79; 95% CI: 0.47–2.02, *p* = 0.52), although there was a trend towards improved survival for patients receiving front-line CPI treatment. 

### 3.3. Treatment with Immune-Checkpoint Inhibitors Followed by BRAF/MEK-Inhibitors

Among the 37 patients who received front-line CPI therapy followed by BRAF ± MEKi therapy, 22 were alive at the time of analysis with a median follow-up time of 41.0 months. Tumor responses achieved with CPI and subsequently with BRAF ± MEK inhibitors are provided in Table 2 and Table 3. Treatment responses achieved within the overall patient cohort (*n =* 243), from which we have selected patients who received sequential CPI and BRAF ± MEKi therapy, are given in Supplementary Table S4.

The median duration of 1L CPI therapy for patients with front-line CPI followed by BRAF ± MEKi therapy was 3.0 months. Most commonly, treatment was discontinued due to disease progression (56.8%), whereas treatment discontinuation due to AE was noted in 43.2% of cases. The median time to disease progression with front-line CPI therapy was 3.0 months (1.3–4.7 months), which corresponded with the median time from progression to the initiation of BRAF ± MEKi therapy (median: 3.0 months), suggesting that the majority of patients (79.4%) did not have a rapidly progressing disease.

Following the initiation of 2L treatment with BRAF ± MEKi, patients remained progression-free for a median of 5.0 months (1.6–8.4 months). In accordance with this, median duration of BRAF ± MEKi was 4.0 months. Notably, seven patients (18.9%) did not experience tumor progression during 2L treatment with BRAF ± MEKi and in four of them treatment was still ongoing at the time of data-lock.

Among the 37 patients showing disease progression upon front-line treatment, 6 patients (16.2%) presented with rapid disease progression, not allowing for the re-introduction of further treatments in four of them. Rapid disease progression was defined as patients showing progressive disease during front-line treatment and who were unable to complete three months of 2L treatment (similar to the first four cycles of CPI therapy). Conversely, slow disease progression was defined as patients presenting with progressive disease during front-line treatment and who subsequently received 2L therapy for more than three months. In our cohort, we identified ten slow progressors: three patients did not show tumor progression during 2L treatment at all, five remained on 2L treatment despite showing tumor progression and two patients received subsequent treatment rechallenge. 

Notably, median overall survival upon cessation of 1L therapy was significantly longer in patients with slow disease progression than in rapid progressors (median OS: 23.0 vs. 6.0 months; *p* = 0.002). Accordingly, our results revealed that the 31 patients not showing rapid disease progression also had a longer OS than rapid progressors (median OS: 41.0 vs. 6.0 months; *p* < 0.001). Among these, 15 were re-initiated with another treatment line, seven continued second-line treatment despite disease progression, two patients were not being given another treatment line due to the occurrence of adverse events and seven patients did not experience tumor progression.

Concerning subsequent treatment lines, we identified 21 patients who received a re-initiation of either treatment with a total of 30 subsequent treatment lines following the initial application of CPI and BRAF ± MEKi. These treatment lines comprised a rechallenge with BRAF ± MEKi (N = 8), CPI (N = 21) or, the application of chemotherapy (N = 1).

### 3.4. Treatment with BRAF/MEK-Inhibitors Prior to Immune-Checkpoint Inhibitor Therapy

**Figure 2:**
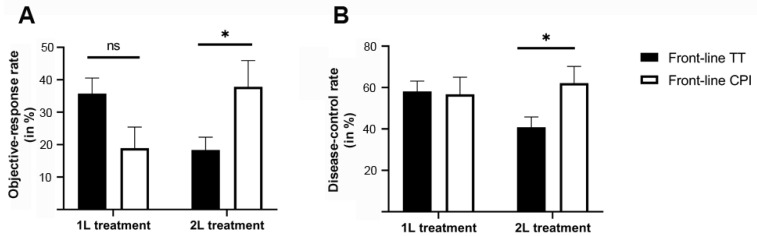
Objective response (**A**) and disease-control rates (**B**) stratified by patients with different treatment sequencing regimens during front-line and second-line treatment. Our results show that patients given front-line CPI therapy had a significantly better objective response (*p* = 0.024, marked with *) and disease-control rate (*p* = 0.034, marked with *) in a second-line setting compared to patients with front-line BRAF ± MEKi therapy. On the other hand, patients with front-line CPI therapy did not have a significantly worse response to first-line treatment with BRAF/MEK-inhibitors (*p* = 0.065 for the comparison of ORR, marked as “ns”; and *p* = 0.92 for DCR), indicating that treatment sequence with front-line CPI might provide better overall response rates. Statistical comparisons of response rates between the different groups were conducted using two-tailed Mann–Whitney test.

Among the 98 patients (47.7%) who received front-line treatment with BRAF ± MEKi followed by CPI therapy, 45 were alive at the time of analysis with a median follow-up time of 43.0 months. Tumor responses achieved with BRAF ± MEKi and subsequently with CPI are provided in Table 2 and Table 3. Given the nonrandomized nature of the retrospective analysis, the different clinicopathological features of the two patient groups and the different indications for BRAF ± MEKi and CPI cross-treatment comparisons in general should be interpreted cautiously. However, using the Chi-square test in order to analyze the treatment outcomes stratified by the treatment sequence, our data indicate that patients with initial BRAF ± MEKi did not show a significantly better response to front-line therapy, with an ORR of 35.7% compared to patients receiving front-line CPI (ORR: 18.4%; *p* = 0.065). On the other hand, the response to 2L CPI was significantly weaker (ORR: 18.9%) compared to 2L BRAF ± MEKi therapy (ORR: 37.8%; *p* = 0.024) (Figure 2).

The median duration of 1L BRAF ± MEKi therapy was 6.0 months. All patients with front-line BRAF ± MEKi therapy experienced disease progression and the median time to disease progression with front-line BRAF ± MEKi therapy was 6.0 months (median PFS; 95% CI: 4.9–7.1 months), which was substantially longer compared to front-line CPI therapy (median PFS: 3.0 months; 95% CI: 1.3–4.7; *p* = 0.020; see Supplementary Figure S1). However, for patients given front-line BRAF ± MEKi, disease progression itself was more rapidly, which corresponded with the median time from progression to the initiation of 2L therapy (median: 1.0 months vs. 3.0 months for front-line CPI, *p* = 0.09). In particular, it was found that among the 98 patients showing tumor progression upon initial BRAF ± MEKi therapy, 87 patients (87.8%) also presented with tumor progression during 2L therapy. Of these, 27 patients (27.6%) presented with rapid disease progression not allowing for more than four cycles of immunotherapy and in 24 patients re-initiation of another treatment line was not possible (88.7%). These rapid progressors showed a median overall survival upon cessation of 1L therapy of only 4 months (95% CI: 1.5–6.4 months), as opposed to slow progressors, with a median overall survival of 51.0 months (95% CI: nA; *p* < 0.001), and a median OS of 42.0 months (10.6–73.4 months) for all patients not showing rapid disease progression upon front-line treatment (see Table 4). The best predictors for rapid disease progression upon front-line treatment were elevated LDH-levels at baseline.

Overall, patients receiving 2L CPI therapy remained progression-free for a median of 2.0 months (95% CI: 1.1–2.9 months), which was considerably shorter than patients with front-line CPI (median PFS: 6.0 months, *p* = 0.019). In accordance, median duration with CPI was only 3.0 months, and 43 patients were not able to complete 3 months of treatment (43.8%). Notably, 11 patients (11.2%) did not experience tumor progression during 2L treatment with CPI and in 5 patients, treatment was still ongoing at the time of data-lock. Among those with tumor progression during 2L CPI therapy, 41 patients received subsequent tumor treatment with an overall number of 70 treatment lines, which comprised the re-introduction of BRAF ± MEKi (*n =* 39), CPI (*n =* 29) or chemotherapy (*n =* 2). 

### 3.5. Impact of the Treatment Sequencing with BRAF/MEK Inhibitors and CPI on Survival in BRAF-Mutant Melanoma Patients

**Figure 3:**
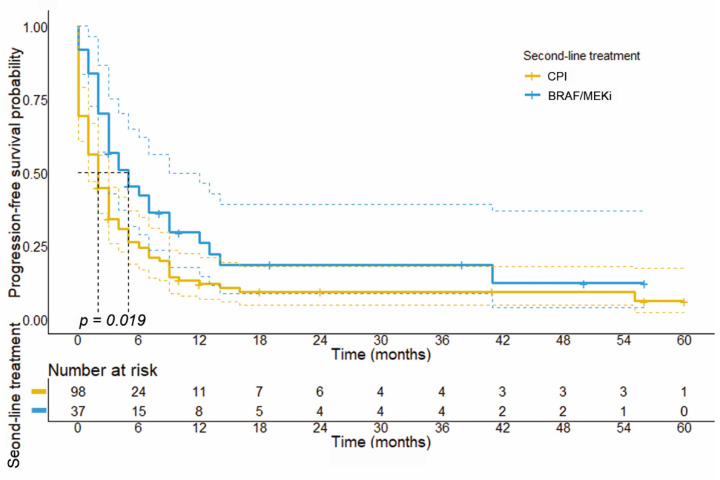
Progression-free survival stratified by the applied treatment in the second-line setting. Results from statistical survival analysis using log-rank test revealed that median PFS of patients with front-line CPI therapy was significantly longer compared to patients with front-line BRAF ± MEKi therapy (median PFS: 5.0 months, 95% CI: 1.6–8.4 vs. 2.0 months, 95% CI: 1.1–2.9; *p* = 0.019).

After analyzing the response to BRAF ± MEKi and CPI stratified by the different treatment schedules, we further explored the impact of treatment sequence on survival. With regard to PFS, Kaplan–Meier plots revealed that patients receiving front-line BRAF/MEKi therapy had a significantly longer PFS (median PFS: 6.0 months; 95% CI: 4.9–7.1 months, *p* = 0.020) compared to patients receiving front-line CPI (median PFS: 3.0 months; 95% CI: 1.3–4.7 months). On the other hand, patients who received CPI following front-line BRAF ± MEKi showed a significantly shorter median PFS (median PFS: 2.0 months; 95% CI: 1.1–2.9 months, *p*= 0.019) compared to patients with BRAF ± MEKi therapy after front-line CPI therapy (median PFS: 5.0 months; 95% CI: 1.6–8.4 months) (see Figure 3). 

**Figure 4:**
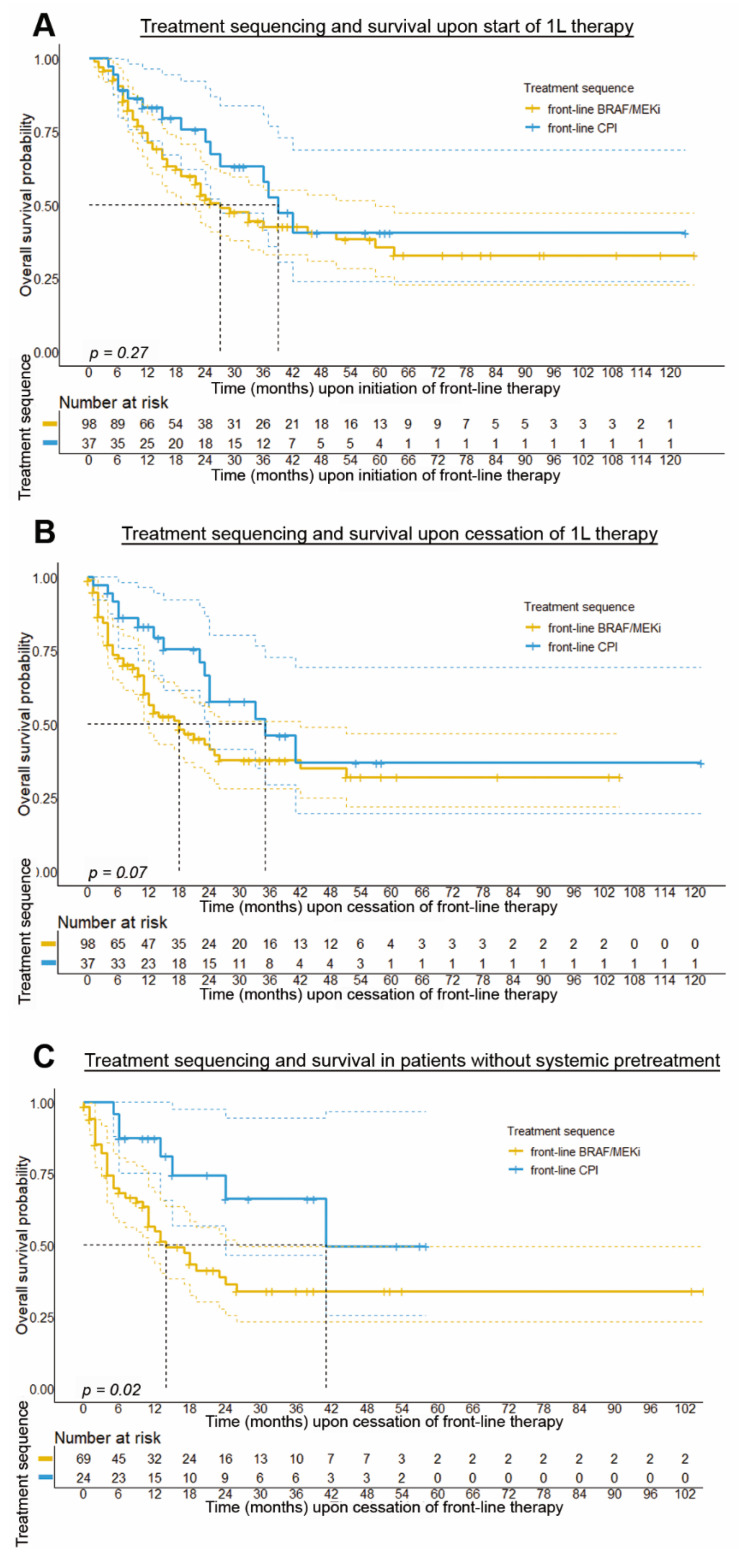
**Overall survival of patients stratified by the treatment regimen applied.** Survival curves show the results of Kaplan–Meier analysis with median OS as primary endpoint, which has been calculated both from initiation of 1L therapy (**A**) and after cessation of 1L therapy (**B**,**C**). Results from subsequent statistical survival analysis using log-rank test reveal that patients who received front-line CPI followed by BRAF ± MEKi therapy showed no statistically significant differences in median OS when calculated from initiation of 1L therapy (median OS: 39.0 vs 29.0 months, *p* = 0.269; (**A**)) or upon cessation of front-line treatment (median OS: 35.0 vs 18.0 months, *p* = 0.070; (**B**)). However, there was a trend towards a better median OS for patients given front-line CPI followed by BRAF ± MEKi therapy. In a sub-cohort of patients without systemic pretreatment (**C**) front-line CPI treatment was associated with a significant survival benefit (median OS: 41.0 vs 14.0 months, *p* = 0.020).

Due to the nonrandomized nature of our analysis, and the limitations in PFS-analysis for treatments being applied in different indications, we also analyzed the OS, stratified by the applied treatment sequence of BRAF ± MEKi and CPI. In accordance with the higher number of rapid disease progressors in the patient group with front-line BRAF ± MEKi therapy, results from our analysis showed that patients with front-line CPI therapy followed by BRAF/MEKi tended to have a longer OS (median OS: 39.0 months; 95% CI: 31.9–46.1 months, *p* = 0.269) compared to patients with front-line BRAF ± MEKi therapy (median OS: 27.0 months, 95% CI: 17.9–36.1 months), albeit this association was below statistical significance (see Figure 4A). This trend became stronger when calculating the median OS upon cessation of 1L therapy (median OS: 35.0 months vs. 18.0 months, *p* = 0.070, Figure 4B).

Of note, further subgroup analysis confirmed the trend that patients with front-line CPI followed by BRAF ± MEKi therapy presented with a longer median overall survival. In particular, it was found that patients who had not received previous adjuvant therapy showed a significantly longer OS for front-line treatment with CPI followed by BRAF ± MEKi (median OS: 41.0 vs. 14.0 months, *p* = 0.02; see Supplementary Table S5 and Figure 4C). In addition, our subgroup analysis indicated that combined CPI therapy was associated with better survival for both sequencing groups, albeit no significant differences in survival could be observed between the different treatment sequence regimens (see Supplementary Table S5).

### 3.6. Response to Subsequent Treatments and Re-Induction of CPI Therapy

Tumor progression upon 2L treatment required the re-initiation of either BRAF ± MEKi or CPI as the third- or fourth-line therapy in 62 patients.

In 45 patients receiving a rechallenge with CPI, renewed tumor control was achieved in 13 patients (28.9%), with 1 patient even presenting with a complete response that was ongoing at the time of data-lock. Interestingly, most of these patients had not shown a response to CPI therapy previously (*n =* 10/13), which indicated that the sequence of BRAF ± MEKi and CPI might have improved the receptivity to immune-checkpoint blockade. The average time from the first application of CPI until a rechallenge of CPI was 9.4 months. CPI-rechallenge included treatment with either combined checkpoint blockade (*n =* 18), anti-PD1 monotherapy (*n =* 26), or anti-CTLA4 monotherapy (*n =* 1), and the median duration of CPI rechallenge was 3.0 months. The overall response rate to CPI rechallenge was 28.9%. However, the median progression-free survival after rechallenge was only 3.0 months (95% CI: 2.2–3.8 months), with no significant differences found for patients with different previous treatment schedules. Notably, a rechallenge with CPI was significantly more often initiated in patients with front-line CPI therapy (*n =* 18; 48.6%) than patients with front-line BRAF ± MEKi therapy (*n =* 27; 27.6%, *p* = 0.025). The median overall survival after CPI rechallenge was 16.0 months (95% CI: 0–36.7 months).

On the other hand, 38 patients received a rechallenge with BRAF ± MEKi. Among these 38 patients, 13 achieved a renewed tumor response (34.2%), with 3 patients even showing a complete response to BRAF ± MEKi rechallenge, which was ongoing in two patients at the time of data-lock. Notably, all patients with a renewed response to BRAF ± MEKi rechallenge received targeted therapy in a front-line setting and have shown at least PR as BOR in nine cases. The median duration of BRAF ± MEKi rechallenge was 6.0 months, and median progression-free survival was 7.0 months (95% CI: 2.7–11.3 months). Overall survival from re-initiation of BRAF/MEKi therapy was 19.0 months (95% CI: 0–46.1 months).

## 4. Discussion

Over the last decade, new systemic treatment options have emerged for treating patients with metastatic melanoma who harbor a BRAF-V600 mutation. The introduction of CPI and BRAF/MEK-inhibitors led to a significant advancement in melanoma therapy, and even profound tumor responses can be found in some patients with advanced melanoma [2,3]. However, despite the promising survival data, accumulating evidence from real-world investigations suggests that most patients with metastatic melanoma harboring a BRAF-mutation will at some timepoint show a relapse of the disease, which requires the re-initiation of anti-tumor treatments due to primary or acquired tumor resistance [9]. Since head-to-head trials comparing the different treatment regimens have not yet been published, it remains a vital issue for cutaneous oncologists to determine the most suitable first-line therapeutic option and the optimal treatment sequencing of BRAF/MEKi therapy and CPI for BRAF-mutant melanoma patients.

In this real-world, retrospective analysis, we present the outcomes of 135 patients with stage IV BRAF-mutated melanoma who received consecutive treatment with BRAF/MEKi and CPI, or vice versa. These were selected from a cohort of 243 melanoma patients who received TT or CPI in a 1L setting (data on a subcohort of patients that only received CPI or BRAF/MEKi therapy without subsequent vice versa treatment are reported in Supplementary Table S7). Here, we obtained several relevant findings that give new insights into the role of treatment sequencing in melanoma therapy and the outcome of BRAF-mutant melanoma patients upon primary treatment failure.

First, our data revealed that the vast majority of the 243 BRAF-mutant melanoma patients receiving front-line BRAF ± MEKi therapy or CPI therapy eventually showed a relapse of the disease within the median follow-up period of 41 months. Notably, patients receiving front-line CPI therapy were less likely to experience a relapse of the disease (83.3% vs. 92.1%) and had a longer median overall survival compared to patients given front-line TT (42.0 months vs 25.0 months, *p* = 0.032) (see Supplementary Table S4). These data are in accordance with a previous retrospective analysis [9], while previous RCT demonstrated superior PFS rates upon 1L therapy with either treatment [5,8]. The different rates of tumor progress upon 1L treatment compared to previous RCT might be explained by the presence of unfavorable prognostic features within our real-world patient cohort, such as a significant share of patients with MBM and elevated LDH serum levels at baseline, which have been excluded from RCT. In addition, differences in median OS and tumor progress might be inferred from the different follow-up periods upon 1L treatment in the overall patient cohort (Supplementary Table S4), and presumably worse prognostic features found for patients receiving front-line TT.

Further, we observed that the 138 melanoma patients with primary treatment failure upon front-line treatment showed a diminished ORR and PFS compared to previous RCT. In particular, we observed a significantly shorter PFS of only 6 months upon front-line TT, as opposed to the COMBI-d and co-BRIM trials, which reported a median PFS of 11–13 months [5]. Moreover, in our patient cohort, only 22.4% of BRAF-mutated patients were progression-free after 12 months of front-line TT compared to >70% in the COMBI-d and co-BRIM trial. The selection bias may most likely explain these data in our patient cohort because we included only patients who showed a tumor relapse upon front-line therapy and subsequently received second-line treatments. Further confounders to be considered are the application of BRAFi-monotherapy in the years 2011–2014 as opposed to combination TT, previous systemic treatments, a significant proportion of patients with multifocal metastatic disease and elevated LDH-serum levels at baseline, and patients with active brain metastasis, who are regularly excluded from clinical trials. Accordingly, a remarkable 70% of patients treated with front-line TT presented with elevated LDH-levels at baseline, and almost 40% showed hepatic metastasis—another detrimental prognostic factor. Thus, it was not unexpected that objective response rates in our patient cohort were considerably weaker (ORR: 35.7%) than previous RCT, which reported response rates between 64 and 69% [5,12]. Therefore, our reported data instead reflect the efficacy of TT in a routine clinical setting for patients with primary treatment failure, which is in accordance with previous studies from real-world cohorts [9,25]. Moreover, it was previously reported that individuals with high LDH levels and brain metastasis have the lowest 1-year PFS (8%) during TT, which highlights the significance of our observations in this patient cohort with adverse prognostic factors [11].

Comparable to the ORR and PFS in patients treated with first-line TT, we observed significantly worse response rates and PFS outcomes for patients with front-line CPI than previous RCTs, namely Keynote-001 and CheckMate067. In particular, we observed a median PFS of only 3 months in our patient’s cohort treated with front-line CPI compared to a reported PFS of 5.6 months upon monotherapy with pembrolizumab [26] and 16.8 months for combined immunotherapy [8]. In addition, we found that only 18% of patients showed an objective response to front-line CPI, which was considerably weaker than previous pooled analysis of nivolumab trials (29.7%) [27]. Here, the worse baseline characteristics found in our patient cohort, and the inclusion of patients having received IPI monotherapy, might account for these significant differences in response and PFS. Further, 24% of patients had received systemic pre-treatments prior to front-line CPI, which might have affected response to front-line treatment.

Despite the discouraging efficacy data of front-line treatment observed in our cohort of BRAF-mutant melanoma patients, overall survival in our cohort was comparable to previous RCTs, with a median OS of 33.0 months compared to 45.5 months for first-line pembrolizumab monotherapy [26] and a median OS of 25.9 and 33.6 months being reported for COMBI-d [5] and the COLUMBUS trial [7], respectively. Therefore, we reasoned that these data might not least be attributed to the availability of second-line treatment options and that treatment sequencing might impact OS.

To test this assumption, we stratified patients according to the applied treatment sequence. In doing so, we observed, second and most importantly, that patients treated with front-line CPI achieved a prolonged OS compared to patients receiving front-line TT (median OS: 35.0 vs. 18.0 months, *p* = 0.07), albeit this association was below statistical significance. Notably, this association became significant in a subgroup of patients without previous systemic treatments (median OS: 41.0 vs. 14.0 months, *p* = 0.02). Our results corroborate previous findings from retrospective studies that reported that front-line CPI treatment resulted in better survival outcomes than front-line TT [9,22,28,29,30] and preliminary data from prospective clinical trials [31]. At the same time these older analyses did not address up-to-date sequences used in melanoma treatment and had substantially shorter follow-up times than our study (see Table 5). Moreover, Schilling and coworkers reported favorable survival outcomes of patients treated with anti-PD1 monotherapy compared to first line TT [16]. In line with this, Moser and coworkers [32] have more recently reported favorable survival outcomes of patients treated with anti-PD1 monotherapy compared to first-line TT, while a small study by Luke and coworkers found no survival benefit for patients receiving front-line CPI [33].

Concerning treatment efficacy in the course of tumor progression, we observed that the DCR was comparable for patients receiving TT prior or after previous CPI exposure (58.2% vs. 62.2%, *p* = 0.84). In comparison, patients receiving front-line CPI had superior DCR to patients receiving CPI after prior application of TT (56.8% vs. 40.8%, *p* = 0.041). Again, these findings are in line with previous studies, which have suggested that BRAF/MEKi therapy efficacy is not substantially influenced by prior CPI [28,35] and that TT maintains a high efficacy throughout different treatment lines, both in the second-line and subsequent treatment lines [36]. Hence, patients who fail CPI may still derive significant benefits from subsequent TT, although, to date, it cannot be ruled out that cross-resistance to TT might exist in patients who initially fail on CPI treatment. Notably, all patients in our study who received a rechallenge with BRAF/MEKi in a third-line setting showed a similar ORR compared to previous treatment lines (ORR: 34.2%), whereas only patients with previous response to BRAF/MEKi therapy were among those with an objective response. In contrast, patients who did not respond to TT rarely responded to CPI, which indicates that the efficacy of CPI therapy might be influenced by prior treatment responsiveness to BRAF/MEKi [21,28]. In line with this, it has been suggested that anti-PD1 is less effective after prior TT than when given in a treatment-naïve setting, both with regard to ORR (33.4% vs. 23.7%) and PFS (median PFS: 7.0 vs. 2.8 months) [26,27,34,37]. Contrasting these reports, a pooled analysis of phase II/III trials could find no evidence that response rates to CPI might be affected by prior TT [27,38].

Last, we showed that survival outcomes upon discontinuation of front-line treatment were substantially worse for patients receiving front-line TT compared to front-line CPI (median OS: 18.0 vs. 35.0 months; *p*= 0.070) and that rapid disease progression was more common in patients with front-line TT (27.6% vs. 16.2%). Furthermore, upon failure of TT, rapid disease progressors only showed a median OS of 4 months [21,28]. These observations are in accordance with retrospective analyses reporting that rapid disease progressors had significantly worse outcomes than patients who were able to complete second-line treatment with ipilimumab [9,20,22,23,28,29,34]. Further, our results confirmed a strong association of elevated LDH levels with rapid disease progression. These data suggest that, in the case of primary failure of TT, the course of the disease might be more aggressive and that patients are thus unable to gain full benefit from subsequent CPI, resulting in rapid PD and death [29]. Considering these results, a recent study of Reijers and coworkers suggested that switching from TT to CPI during an ongoing response to TT might be superior in terms of survival compared to switching from TT to CPI at the moment of PD [20]. This suggestion is supported by preclinical studies which showed a favorable transformation of the TME during initial BRAF-inhibitor therapy, while demonstrating that T cell infiltration was diminished at the time of disease progression [39,40], a question that is currently being addressed in the prospective clinical trial SECOMBIT (Supplementary Table S6). 

Significant limitations of our study are the retrospective, monocentric nature of the investigation, which generates an inherent selection bias within the cohort. Our real-life data also show an accumulation of patients with unfavorable baseline characteristics among those receiving first-line TT, which might have affected our analysis, despite correction with multivariate Cox-regression analysis. Additionally, due to the overrepresentation of patients with poor prognostic features and front-line treatment with TT, the sample size was not balanced in the groups comparing the OS in patients receiving CPI before TT or CPI after TT. Therefore, survival benefits found in patients with front-line CPI treatment might also be inferred, either from the poor prognostic features found in the cohort which received front-line TT, or the observation that some patients with 1L CPI had initially discontinued CPI due to severe AE and only later due to PD. Moreover, the heterogeneity in terms of the different classes of CPI and BRAF/MEKi administered, the different systemic pre-treatments and subsequent treatment lines, as well as the different standards of treatments applied (i.e., therapy with single-agent BRAF-inhibitors or ipilimumab was standard schema in the years 2011–2014) might have affected our results. In particular, subgroup analysis found that patients who received combined checkpoint-inhibitor therapy showed substantially better survival data than patients with CPI monotherapy. We also acknowledge that, by studying patients who received second-line treatment, we introduced a selection bias because only patients who were fit enough would start second-line therapy. To examine the impact of subsequent therapies on treatment outcomes, randomized controlled trials with predefined endpoints are required, since, in real-world cohorts, the choice of subsequent therapy depends on tumor characteristics and the preference of the treating oncologist. Finally, there was no homogenous timing and frequency of response assessments, making PFS data susceptible to confounding, while OS is less likely to be affected and was therefore defined as a primary endpoint. Given the above-discussed limitations, our data need to be interpreted cautiously. 

In summary, the results of this retrospective analysis indicate that front-line CPI therapy followed by BRAF/MEKi therapy might provide better tumor control in the long-run, albeit we could only show a significant association with OS in a subgroup of previously untreated patients and not in the overall cohort of patients with BRAF-mutant, metastatic melanoma. Our results also show that survival was inferior for patients who were refractory to BRAF/MEKi and that these patients were at higher risk of rapid disease progression than patients with upfront CPI therapy. Importantly, our real-world data suggest that this association might even apply to patients with adverse prognostic features, such as melanoma brain metastases and high LDH levels. However, these results require further validation in prospective clinical trials, which are currently underway.

## 5. Conclusions

This retrospective study, including 135 metastatic melanoma patients who received consecutive treatments with BRAF/MEKi and CPI, or vice versa, provides evidence that front-line treatment with CPI is associated with favorable tumor control and overall survival in patients with BRAF-mutant melanoma. Further, our data indicate that patients who are refractory to front-line BRAF/MEKi therapy are at higher risk of rapid disease progression compared to patients with front-line CPI treatment.

## Figures and Tables

**Table 1:** Baseline patient characteristics and treatment outcomes.

Clinical-Pathological Features	BRAF ± MEKi Prior to CPI (Group 1)	BRAF ± MEKi after CPI (Group 2)	*p*-Value
Overall number of patients	98	37	
Median age at initiation of 1L treatment	58.5 years (26–86)	60.0 years (32–81)	0.184
Gender			0.503
female	44 (44.9%)	19 (51.4%)	
male	54 (55.1%)	18 (48.6%)	
Primary tumor and metastasis			
Median Breslow thickness ^1^ (range)	2.3 (0.6–35.0 mm)	2.15 (0.6–6.0 mm)	0.171
Ulceration ^2^	37/59 (62.7%)	9/25 (36.0%)	0.032
Elevated serum LDH levels (>245 U/L) ^3^	46/63 (73.0%)	22/31 (71.0%)	0.509
Melanoma brain metastasis	52 (53.1%)	17 (45.9%)	0.563
Liver metastasis	40 (40.8%)	9 (24.3%)	0.056
**1L Therapy, n (%)** cICB (IPI + Nivo) Anti-PD1: - nivolumab - pembrolizumab Anti-CTLA4 (IPI) BRAFi or MEKi monotherapy - vemurafenib - dabrafenib BRAF/MEKi therapy - dabrafenib + trametinib - vemurafenib + cobimetinib - encorafenib + binimetinib	0 (0%) 0 (0%) 0 0 0 (0%) 38 (38.8%) 29 (29.5%) 9 (9.2%) 60 (61.2%) 46 (46.9%) 13 (13.3%) 1 (1.0%)	17 (45.9%) 14 (37.8%) 7 (18.9%) 7 (18.9%) 6 (16.2%) 0 (0%) 0 (0%)	
Previous systemic treatments	42/65 (31.1%)	13/37 (24.3%)	
Median duration of 1L therapy (range)	5.0 months (1–87)	3.0 months (1–22)	0.020
Overall response to 1L therapy ^4^	35/98 (35.7%)	7/37 (18.9%)	0.065
Progress during 1L therapy	98/98 (100%)	37/37 (100%)	
Median progression-free survival upon 1L therapy (95% CI)	6.0 months (4.8–7.2)	3.0 months (1.3–4.7)	0.025
Time-to-next-treatment in months (range)	1.0 (0–56)	3.0 (0–32)	0.090
**2L therapy** cICB (IPI + Nivo) Anti-PD1: - nivolumab - pembrolizumab Anti-CTLA4 (IPI) BRAF or MEKi monotherapy - vemurafenib - dabrafenib - trametinib BRAF/MEKi therapy - dabrafenib + trametinib - encorafenib + binimetinib Overall response to 2L therapy ^5^ Median treatment duration 2L therapy Progress during 2L therapy Median progression-free survival upon 2L therapy (95% CI)	41 (41.8%) 29 (29.6%) 12 (12.2%) 17 (17.3%) 28 (28.6%) 0 0 17/98 (18.4%) 3.0 months (0–41) 87/98 (88.8%) 2.0 months (1.1–2.9)	0 0 0 8 (21.6%) 2 (5.4%) 3 (8.1%) 3 (8.1%) 29 (78.4%) 22 (59.5%) 7 (18.9%) 14/37 (37.8%) 4.0 months (1–38) 29/37 (78.4%) 5.0 months (1.6–8.4)	**0.024** **0.009** 0.164 **0.019**
**Subsequent treatment lines** Patients receiving 3L therapy Overall number of all subsequent (3L+) therapy lines BRAF ± MEKi therapy CPI therapy Other	41 (41.8%) 70 39 (55.7%) 29 (41.4%) 2 (2.9%)	21 (56.8%) 30 8 (26.6%) 21 (70.0%) 1 (3.3%)	0.253 0.373
Median follow-up upon initiation of 1L treatment (95% CI) Median overall survival following 1L therapy initiation (95% CI)	43.0 months (27.4–58.6) 27.0 months (17.9–36.1)	41.0 months (28.2–53.8) 39.0 months (31.9–46.2)	0.159 0.269
Median overall survival upon cessation of 1L therapy (95% CI)	18.0 months (9.5–26.4)	35.0 months (17.9–52.1)	0.070
Deceased	53 (54.1%)	15 (40.5%)	0.181

Abbreviations: CR = complete response; PR = partial response; SD = stable disease; PD = progressive disease, ORR = objective response rate; CPI = immune checkpoint inhibitors; CI = confidence interval; cICB = combined checkpoint-inhibitor blockade; IPI = ipilimumab; nivo = nivolumab; pembro = pembrolizumab; TP = tumor progression; ^1,2,3^ Statistics based on the total number of patients with known Breslow thickness (*n =* 101), ulceration status (*n =* 84) and LDH serum levels (*n =* 94). ^4^ Statistics based on the total number of patients with known BOR to 1L therapy with BRAF/MEKi or CPI (*n =* 132); ^5^ Statistics based on the total number of patients with known response to 2L therapy (*n =* 131). The *p*-value is indicated in bold numbers when statistically significant.

**Table 2:** Response to front-line treatment in patients with consecutive treatment with CPI and TT stratified by the sequence regimen investigated.

Outcome	Response to Front-Line BRAF ± MEKi Therapy (Group 1)	Response to Front-Line CPI Therapy (Group 2)	*p*-Value
Best overall response (%)			0.120
Complete response (CR)	6 (6.1%)	- (0.0%)	
Partial response (PR)	29 (29.6%)	7 (18.9%)	
Stable disease (SD)	22 (22.4%)	14 (37.8%)	
Progressive disease (PD)	41 (41.8%)	16 (43.2%)	
Objective-response rate (ORR)			0.065
Number (%)	35/98 (35.7%)	7/37 (18.9%)	
95% CI ^1^	26.3–46.0%	8.0–35.2%	
Disease control rate (DCR)			0.92
Number (%)	57/98 (58.2%)	21/37 (56.8%)	
95% CI ^1^	47.8–68.1%	39.5–72.9%	
Progress during 1L therapy			1.0
Number (%)	98/98 (100%)	37/37 (100%)	
95% CI ^1^	94.4–100%	90.3–100%	

Abbreviations: Objective response rate was defined as the percentage of patients who obtained CR or PR; disease control rate was defined as the percentage of patients who obtained CR, PR, or SD. ^1^ The 95% confidence intervals were calculated using the Clopper–Pearson method.

**Table 3:** Response to second-line treatment in patients with consecutive treatment with CPI and TT stratified by the sequence regimen investigated.

Outcome	Response to 2L CPI after Initial BRAF ± MEKi Therapy (Group 1)	Response to 2L BRAF ± MEKi Following Initial CPI Therapy (Group 2)	*p*-Value
Best overall response (%)			0.068
Complete response (CR)	3 (3.2%)	2 (5.6%)	
Partial response (PR)	15 (15.3%)	12 (32.4%)	
Stable disease (SD)	22 (22.4%)	9 (23.0%)	
Progressive disease (PD)	58 (59.2%)	14 (37.8%)	
Could not be assessed	0	0	
Objective-response rate (ORR)			**0.024**
Number (%)	18/98 (18.4%)	14/37 (37.8%)	
95% CI ^1^	11.3–27.5%	22.5–55.2%	
Disease control rate (DCR)			**0.034**
Number (%)	40/98 (40.8%)	23/37 (62.2%)	
95% CI ^1^	31.0–51.2%	44.8–77.5%	
Progress during 1L therapy			0.164
Number (%)	87/98 (88.8%)	29/37 (78.4%)	
95% CI ^1^	80.8–94.3%	61.8–99.1%	

Abbreviations: Objective response rate was defined as the percentage of patients who obtained CR or PR; disease control rate was defined as the percentage of patients who obtained CR, PR, or SD. ^1^ The 95% confidence intervals were calculated using the Clopper–Pearson method.

**Table 4:** Correlation between baseline factors and slow and rapid disease progression.

Patient Characteristics, n (%)	Non-Rapid Progressors (*n =* 102)	Rapid Progressors (*n =* 33)	*p*-Value
Previous treatments	31 (230.4%)	11 (33.3%)	0.454
Elevated LDH ^1^	50 (67.6%)	18 (90.0%)	**0.038**
MBM	51 (50.0%)	18 (54.5%)	0.104
Hepatic metastasis	35 (34.3%)	14 (42.4%)	0.261
Gender, male	50 (49.0%)	22 (66.6%)	0.108
Age, years	57.5 (26–81)	62.0 (28–86)	0.348
BRAF ± MEKi prior to CPI	71 (72.4%)	27 (27.6%)	0.188
BRAF/MEKi after CPI	31 (83.8%)	6 (16.2%)	-
Median overall survival following cessation of 1L treatment			
All patients (*n =* 135)	41.0 months (16.2–65.7)	4.0 months (1.9–6.1)	**<0.001**
BRAF ± MEKi prior to CPI	42.0 months (10.6–73.4)	4.0 months (1.5–6.4)	**<0.001**
BRAF ± MEKi after CPI	41.0 months (28.9–53.1)	6.0 months (4.3–7.7)	**<0.001**

Abbreviations: ^1^ known LDH serum levels of non-rapid progressors (*n =* 74) and rapid progressors (*n =* 20).

**Table 5:** Comparison of outcomes reported in different retrospective trials for patients with BRAF-mutant stage IV melanoma stratified by treatment sequence.

Authors	No. of Patients	Elevated LDH at 1L Therapy Initiation	MBM	ORR to 2L CPI (%)	ORR to 2L TT (%)	Median PFS upon 2L TT, Months	Median PFS upon 2L CPI, Months	Median OS (Months) upon Initiation of 1L TT	Median OS (Months) upon Initiation of 1L CPI	Median Follow-Up Time
Ascierto [22]	93	nA	nA	10%	nA	nA	nA	9.9 months	14.5 months	11 months
Ackerman [28]	274	34%	20%	0%	57%	nA	2.7	13.4 months	19.6 months	nA
Johnson [23]	114	40% (TT) vs. 19% (CPI)	24% (TT) vs. 9% (CPI)	25%	nA	2.8 months	5.0 months	40.3 months	27.5 months	nA
Amini-Adle [34]	74	46.3%	34.1%	12.2%	nA	nA	2.0 months	nA	7.0 months (from start anti-PD1)	nA
Czarnecka [9]	253	42.3%	24.9%	8%	nA	12.7 months	2.4 months	11.7 months	not reached (14.7—NR)	23.2 months
Simeone [29]	47	52.3%	21.4%	12.5%	nA	9 months	3 months	nA	nA	nA
Haist et al	135	72.3%	51.1%	18.4%	37.8%	5.0 months	2.0 months	27.0 months	39.0 months	41 months

Abbreviations: No. = number; 1L = first line; 2L = second line; MBM = melanoma-brain metastases; ORR = objective response rate; PFS = progression-free survival; OS = overall survival; TT = BRAF/MEK-directed targeted therapy; CPI = checkpoint-inhibitor therapy; nA = not available; NR = not reached.

## Data Availability

All relevant data are within the manuscript and its supporting tables and figures. The individual patients’ data is not publicly available and cannot be shared.

## Supplementary Materials

The following supporting information can be downloaded at: https://www.mdpi.com/article/10.3390/cancers14092082/s1. Figure S1: Progression-free survival upon first-line therapy with BRAF/MEKi or CPI, Figure S2: Waterfall plot showing the response durability and objective response to first- and second-line treatment of all 135 patients, Table S1: Definition of real-world endpoints used in this study, Table S2: Univariate Cox proportional hazards model for overall survival, Table S3: Multivariate Cox proportional hazards model for overall survival, Table S4: Response to and survival upon front-line treatment as evaluated in the overall patient cohort, Table S5: Analysis of the median overall survival upon cessation of 1L therapy for different patient subgroups, Table S6: List of current randomized-controlled trials investigating the impact of treatment sequencing on survival outcomes, Table S7: Baseline patient characteristics, treatment specifics and survival outcomes in patients with CPI or BRAF/MEKi therapy only.

## Abbreviations

AE	adverse events
BRAF/MEKi	BRAF/MEK inhibitors
BRAF ± MEKi	BRAF with/or without MEK inhibitors
BSC	best-supportive care
CI	confidence interval
CPI	immune-checkpoint inhibitors
CR	complete response
CTLA-4	cytotoxic-T-lymphocyte antigen 4
DCR	disease-control rate
HR	hazard ratio
ICB	immune-checkpoint blockade
LDH	lactate dehydrogenase
MBM	melanoma brain metastases
ORR	overall response rate
OS	overall survival
PD	progressive disease
PD-1	programmed death protein 1
PFS	progression-free survival
PR	partial response
RCT	randomized controlled trial
SD	stable disease
Tx	treatments
TME	tumor microenvironment
TT	BRAF/MEK-directed targeted therapy
TTNT	time to next treatment
1L	front-line
2L	second-line
