# Supplementary material for: The Role of Treatment Sequencing with Immune-Checkpoint Inhibitors and BRAF/MEK Inhibitors for Response and Survival of Patients with BRAFV600-Mutant Metastatic Melanoma—A Retrospective, Real-World Cohort Study
Source: Cancers (Basel). 2022 Apr 21;14(9):2082. doi: 10.3390/cancers14092082 (PMC9101790; doi:10.3390/cancers14092082)
Supplement: Supplementary file 1 [file cancers-14-02082-s001.zip › cancers-1653989-supplementary.pdf]

cancers

MDPI

Supplementary Tables and Figures

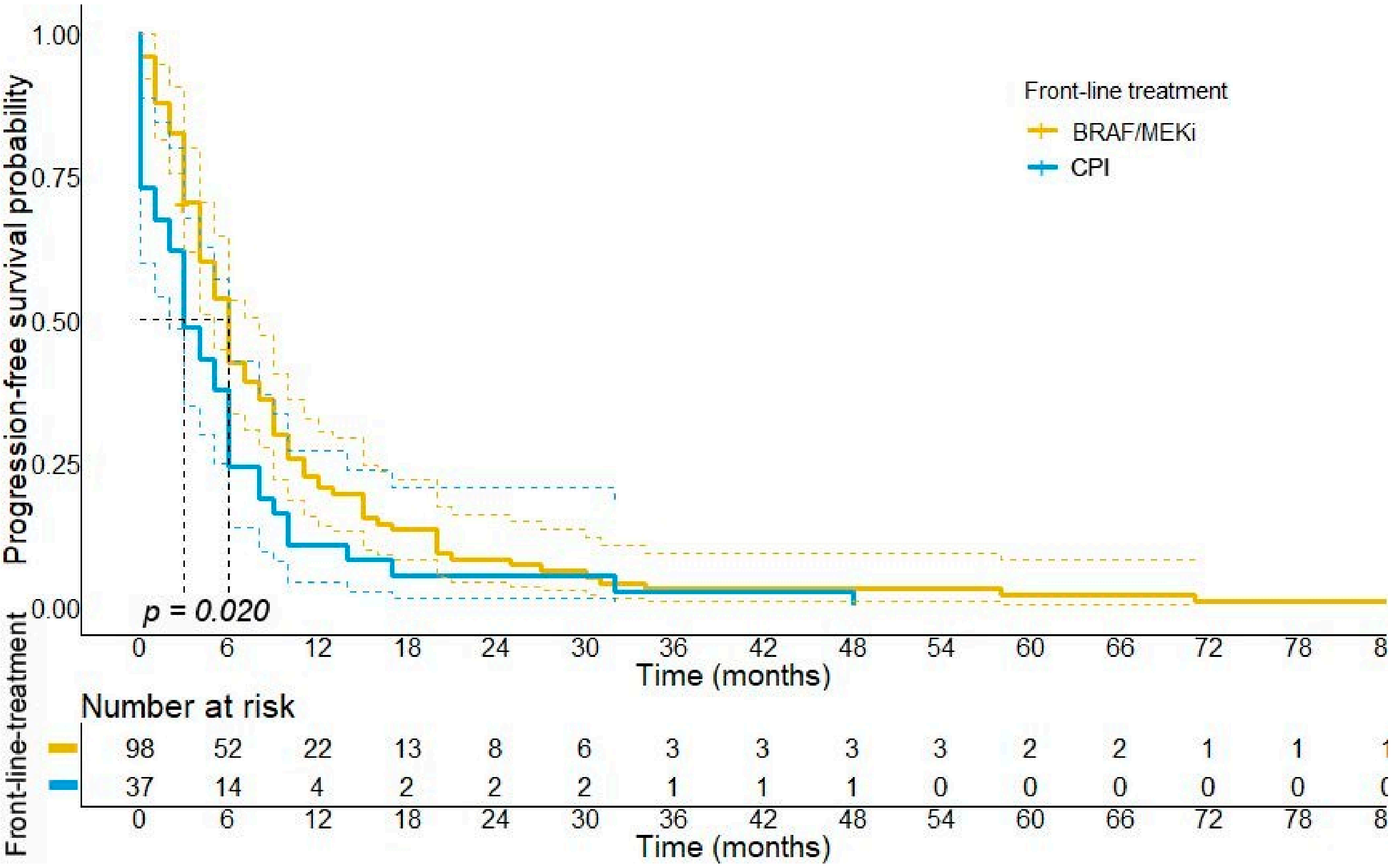

Supplementary Figure S1: Progression-free survival upon first-line therapy with BRAF/MEK-inhibitors or immune-checkpoint inhib- itors (CPI) in patients who received consecutive treatment with either treatment (n=135). Kaplan-Meier analysis revealed a signifi- cantly longer median PFS for front-line BRAF/MEKi therapy in this patient cohort (median PFS: 6.0 vs 3.0 months, p = 0.020)

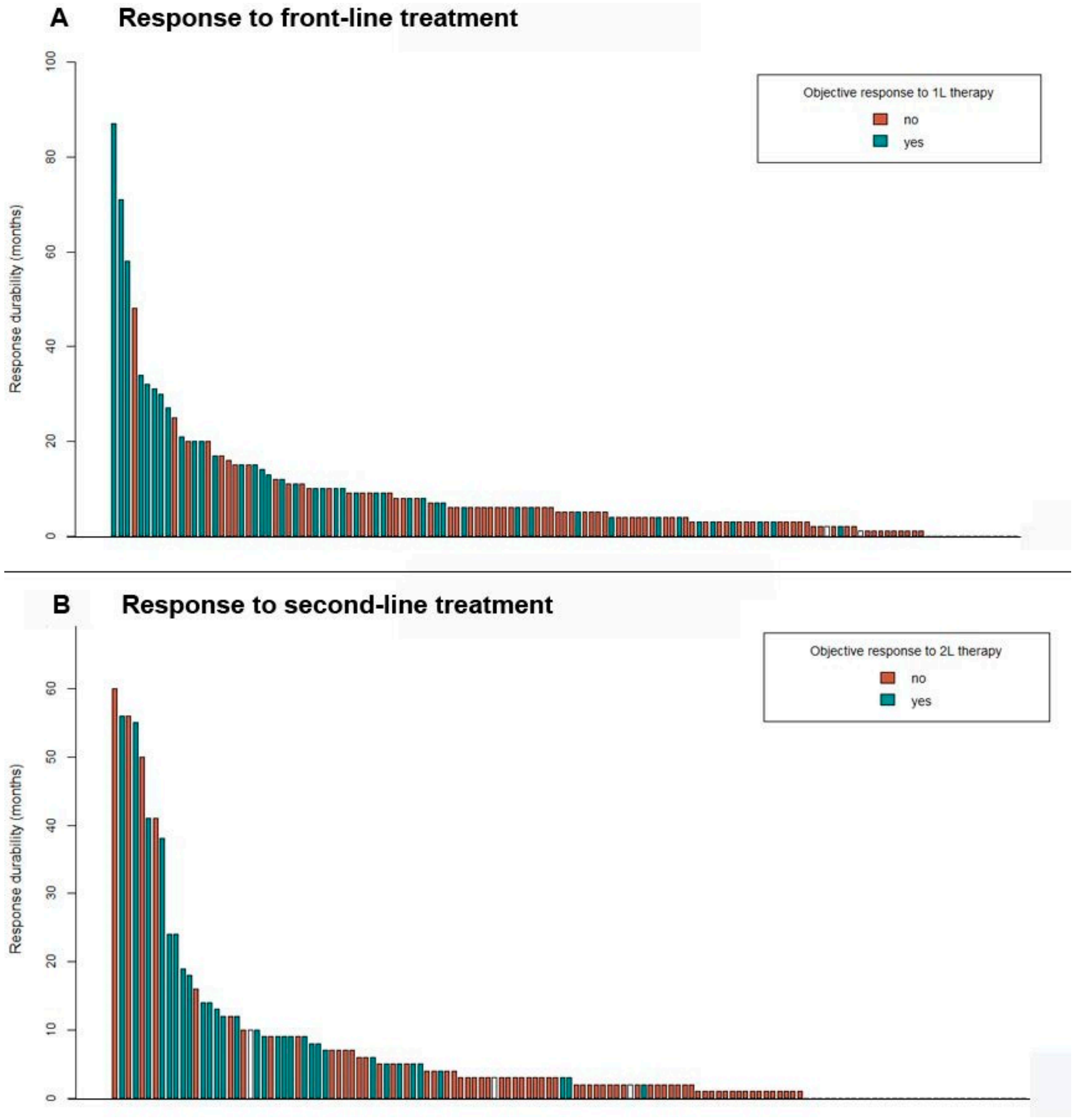

Supplementary Figure S2: Waterfall plot showing the response durability of all 135 patients included in the retrospective analysis and whether patients showed an objective response to front-line or second-line treatment. Response durability was defined as the time from the start of front-line treatment (A) or second-line treatment (B) until first date of disease progression. Objective response was based on the real-world response assessments and was defined as either complete response or partial response to treatment.

Supplementary Table S1: Definition of real-world endpoints used in this study.

Endpoint	Outcome
**Primary**	
Overall survival (OS)	The time interval from index date to date of death. Patients alive at the date of last contact were censored. Index dates were defined as:<br>- start of 1L treatment<br>- end of 1L treatment
**Secondary**	
Best-overall response (BOR)	Best tumor response defined as complete response (CR), partial response (PR), stable disease (SD), or progressive disease (PD) according to the Revised Response Evaluation Criteria in Solid Tumors (RECIST) 1.1. guidelines
Objective response rate (ORR)	The proportion of patients with a complete response or partial response based on real-world response assessments* relative to all patients initiating treatment. (For the best therapy response both the clinical assessments in the medical record and the radiological assessment in the staging findings were being evaluated).
Disease control rate (DCR)	The proportion of patients who had a complete response, partial response, or stable disease based on real-world response assessments*. (For the best therapy response both the clinical assessments in the medical record and the radiological assessment in the staging findings were being evaluated).
Progression-free survival (PFS)	The time interval from index date to physician-reported date of progression, death date or start date of a new treatment due to progression of disease (whichever event occurred first). Patients without a progression event or date of death were censored at the date of last contact.

* Complete response: complete resolution of all visible disease; partial response: disease still present, with partial reduction in size of visible disease in some or all areas without any areas of increase in visible disease; stable disease: no change in overall size of visible disease or mixed response.

Supplementary Table S2: Univariate Cox proportional hazards model for overall survival

Variables	Subgroups	HR	95% CI	*p*-value
Age (years)	>59 vs. ≤59	1.40	0.87-2.26	0.17
Gender	Male vs. female	1.1	0.68-1.77	0.71
Breslow (mm)	>2mm vs. ≤2mm	1.36	0.78-2.39	0.28
Ulceration	Yes vs. No	1.09	0.60-1.98	0.78
LDH serum levels at baseline	Elevated vs. Normal	2.34	1.08-5.1	**0.02**
Hepatic metastasis	Yes vs. No	2.00	1.2-3.3	**0.007**
Brain metastases	Yes vs. No	1.73	1.04-3.21	**0.036**
Previous treatments	No vs. Yes	0.93	0.56-1.55	0.79
BOR to frontline treatment	CR, PR vs. SD, PD	0.36	0.20-0.66	**<0.001**
Duration 1L treatment	> 5 months vs. ≤ 5months	0.35	0.22-0.58	**<0.001**
BOR to second-line treatment	CR, PR vs. SD, PD	0.29	0.14-0.61	**0.001**
Treatment duration 2L	>3 months vs. ≤3	0.26	0.15-0.47	**<0.001**
Treatment sequence	front-line CPI vs. front-line BRAF	0.73	0.41-1.29	0.28

The p value is indicated in bold numbers when statistically significant. Abbreviations: CR = complete response, PR = partial response, SD = stable disease, PD = Progressive disease, BOR = best overall response, HR = hazard ratio, CI = confidence interval; 2L = second-line

Supplementary Table S3: Multivariate Cox proportional hazards model for overall survival

Variables	Subgroups	HR	95% CI	*p*-value
LDH serum levels at baseline	Elevated vs. Normal	2.26	1.02-4.98	**0.044**
Hepatic metastasis	Yes vs. No	2.12	1.03-4.38	**0.040**
Brain metastases	Yes vs. No	1.21	0.59-2.33	0.54
BOR to frontline treatment	CR, PR vs. SD, PD	0.55	0.26-1.13	0.103
BOR to second-line treatment	CR, PR vs. SD, PD	0.34	0.13-0.92	**0.020**

Therapy Sequence	front-line CPI vs. front-line BRAF	0.79	0.47-2.02	0.52

The p value is indicated in bold numbers when statistically significant. Abbreviations: CR = complete response, PR = partial response, SD = stable disease, PD = Progressive disease, BOR = best overall response, HR = hazard ratio, CI = confidence interval; 2L = second-line

Supplementary Table S4: Response to and survival upon front-line treatment as evaluated in the overall patient cohort.

Outcome	Response to front-line BRAF±MEKi therapy (n=177)	Response to front-line CPI therapy (n=66)	*p*-value
Best overall response (%)			0.325
Complete response (CR)	13 (7.3%)	4 (6.1%)	
Partial response (PR)	56 (31.6%)	18 (27.2%)	
Stable disease (SD)	38 (21.4%)	22 (33.3%)	
Progressive disease (PD)	67 (37.9%)	21 (31.8%)	
could not be assessed	3 (1.7%)	1 (1.5%)	
Objective-response rate (ORR)			0.762
Number (%)	64/174 (36.7%)	22/65 (33.8%)	
95% CI[1]	26.3-46.0%	22.6-46.6%	
Disease control rate (DCR)			0.182
Number (%)	100/174 (57.4%)	44/65 (66.7%)	
95% CI[1]	47.8-68.1%	54.9-78.8%	
Progress during 1L therapy			**0.046**
Number (%)	163/177 (92.1%)	55/66 (83.3%)	
95% CI[1]	87.1-95.6%	72.1- 91.4%	
Median OS upon 1L therapy start	25.0 months (15.7-34.3 months)	42.0 months (nA-nA)	**0.032**
Median Follow-up time upon start of 1L therapy	42.0 months (33.0 – 51.0 months)	25.0 months (16.8-33.1 months)	**0.047**

Abbreviations: Objective response rate was defined as the percentage of patients who obtained CR or PR; disease control rate was defined as the percentage of patients who obtained CR, PR, or SD. [1] The 95% confidence intervals were calculated using the Clopper- Pearson method.

Supplementary Table S5: Analysis of the median overall survival upon cessation of 1L therapy for different patient subgroups

	**Median OS upon 1L therapy cessation, months (95% CI)**		
Patient characteristics	BRAF±MEKi prior to CPI (n=98)	BRAF±MEKi after CPI (n=37)	*p*-value
All patients	18.0 months	35.0 months	0.070
Previous treatment			
no	14.0 months (7.3-20.7)	41-0 months (29.7 – NR)	**0.020**
yes	25.0 months (0-56.5)	24.0 months (10.2-37.8)	0.822
Elevated LDH	23.0 months (15.7-30.3)	33.0 months (9.4-56.5)	0.363
MBM	11.0 months (7.6-14.4)	23.0 months (11.9-34.0)	0.169
Hepatic metastases	11.0 months (7.9-14.1)	13.0 months (7.1-18.9)	0.743
CPI monotherapy	18.0 months (10.1-25.8)	35.0 months (18.2-51.7)	0.164
CPI combination therapy	28.8 months (21.1-36.6)	not reached	0.213

Supplementary Table S6: List of current randomized clinical trials investigating the impact of treatment sequencing on survival outcomes in patients with unresectable stage III or metastatic stage IV, BRAF-mutant melanoma.

National Clinical Trial (NCT) number	Trial	Conditions	Interventions	Clinical Phase	Start date
NCT02224781	dabrafenib and trametinib Followed by ipilimumab and nivolumab or ipilimumab and nivolumab Followed by dabrafenib and trametinib in Treating Patients With Stage III-IV BRAFV600 Melanoma (DREAMSeq) [26]	Unresectable stage III, or stage IV, BRAF-mutated melanoma	1. Upfront IPI+Nivo q3w for 2 cycles, followed by Nivo monotherapy until PD; upon PD cross-over to Dab+Tram<br>2. Upfront DabTram therapy until PD; upon PD cross-over to IPI+Nivo q3w for 2 cycles, followed by Nivo monotherapy	Phase III	07/2015
NCT02968303	Phase 2 Study With Combination of vemurafenib With cobimetinib in B-RAF V600E/K Mutated Melanoma Patients to Normalize LDH and Optimize nivolumab and ipilimumab therapy (COWBOY)	Unresectable stage III, or stage IV, BRAF-mutated melanoma	1. 6 weeks of Vem+Cob followed by IPI+Nivo and Nivo q4w<br>2. Upfront IPI+Nivo followed by Nivo q4w, without prior induction with Vem+Cob	Phase II	01/2017
NCT02631447	A Three Arms Prospective, Randomized Phase II Study to Evaluate the Best Sequential Approach With Combo Immunotherapy (ipilimumab/nivolumab) and Combo Target Therapy (LGX818/MEK162) in Patients With Metastatic Melanoma and BRAF Mutation (SECOMBIT)	Unresectable stage III, or stage IV, BRAF-mutated melanoma	1. Enco+Bini until PD, followed by IPI+Nivo q3w and subsequent Nivo q2w until PD<br>2. IPI+Nivo q3w, followed by Nivo q2w until PD, then Enco+Bini<br>3. Enco+Bini for 8 weeks followed by IPI+Nivo q3w and Nivo q2w until PD, then Enco+Bini until PD	Phase II	11/2016

NCT02902029	A Phase II, Open-label, Randomized-controlled Trial Evaluating the Efficacy and Safety of a Sequencing Schedule of cobimetinib Plus vemurafenib Followed by Immunotherapy With an Anti- PD-L1 Antibody Atezolizumab for the Treatment in Patients With Unresec- table or Metastatic BRAF V600 Mutant Melanoma (ImmunoCobiVem)	Unresectable stage III, or stage IV, BRAF-mutated melanoma excluding melanoma brain metastases	1. 3-month run-in period with Vem+Cob followed by Vem+Cob until PD; upon PD cross-over to Atezolizumab 1200mg q3w<br>2. 3-month run-in period with Vem+Cob followed by atezolizumab q3w until PD; upon PD cross-back to Vem+Cob	Phase II	09/2016
NCT03235245	Combination of Targeted Therapy (encorafenib and binimetinib) Followed by Combination of Immunotherapy (ipilimumab and nivolumab) vs Immediate Combination of Immunotherapy in Patients With Unresectable or Metastatic Melanoma With BRAF V600 Mutation: an EORTC Randomized Phase II Study (EBIN)	Unresectable stage III, or stage IV, BRAF-mutated melanoma	1. IPI+Nivo q3w followed by Nivo q4w until completion of 2 years or PD<br>2. Enco+Bini for 12 weeks followed by a pause, and IPI+Nivo q3w and Nivo q4w until completion of 2 years or PD; in case of PD cross-back to Enco+Bini	Phase II	08/2017

Abbreviations: Bini = binimetinib; Cob= cobimetinib; Dab = dabrafenib; Enco = encorafenib; IPI = ipilimumab; Nivo = nivolumab; PD = progressive disease; Tram = trametinib; Vem = vemurafenib;

Supplementary Table S7: Baseline patient characteristics, treatment specifics and survival outcomes in patients with known BRAF V600 mutation and treatment with immune-checkpoint inhibitors or BRAF±MEKi only.

Clinical-pathological features	CPI only	BRAF±MEKi only
Overall number of patients	29	79
Median age at initiation of 1L treatment	67.0 years (33-84)	58.5 years (24-89)
Gender		
female	10 (34.5%)	38 (48.1%)
male	19 (63.3%)	41 (51.9%)
Primary tumor and metastasis		
Median Breslow thickness[1] (range)	1.7 (0.26-7.0mm)	2.93 (0.38-8.4mm)
Ulceration[2]	6/20 (30.0%)	9/20 (45.0%)
Elevated serum LDH levels (>245 U/l)[3]	14/27 (51.8%)	9/11 (81.1%)
Melanoma brain metastasis	6 (20.6%%)	41/73 (56.2%)
Liver metastasis	7 (24.1%)	24/69 (34.7%)
Treatments and Survival		
1L Therapy, n (%)		
cICB (IPI+Nivo)	9 (31.0%)	0
Anti-PD1:	20 (69.0%)	0
- nivolumab	6 (20.7%)	0
- pembrolizumab	14 (48.3%)	0
Anti-CTLA4 (IPI)	0	0
BRAFi or MEKi monotherapy	0 (0%)	33/79 (41.8%)
BRAF/MEKi therapy	0	46/74 (58.2%)
Previous systemic treatments	4/29 (13.8%)	17/65 (26.2%)
Median duration of 1L therapy (range)	7.0 months (1-38)	5.0 months (0-118)
Best-overall response to 1L therapy		
- CR	4 (13.8%)	7 (8.9%)
- PR	11 (37.9%)	27 (34.1%)
- SD	8 (27.5%)	16 (20.3%)
- PD	5 (17.2%)	26 (32.9%)
- nA	1	2 (2.5%)
Overall response to 1L therapy[4]	15/29 (53.6%)	34/77 (44.1%)
Progress during 1L therapy	18/29 (62.1%)	65/79 (82.2%)
Discontinuation due to AE	5/29 (17%)	9/39 (23.01%)
Median progression-free survival upon 1L therapy (95% CI)	11.0 months (1.8-20.2)	5.0 months (3.7-6.3)
2L therapy		
CPI rechallenge	7/29 (24.1%)	0
BRAF/MEKi rechallange	0	9/79 (11.4%)
Median follow-up upon initiation of 1L treatment (95% CI)	21.0 months (16.6-25.4)	42.0 months (5.7-78.3)
Median OS following 1L therapy initiation (95% CI)	NR (NR-NR)	19.0 months (5.7-32.3)

Median OS upon cessation of 1L therapy (95% CI)	NR (NR-NR)	7.0 months (0-15.4)
Deceased	8/29 (27.6%)	39/79 (49.3%)

Abbreviations: CR = complete response; PR = partial response; SD = stable disease; PD = progressive disease, CPI = immune check- point inhibitors; CI = confidence interval; cICB = combined checkpoint-inhibitor blockade; IPI = ipilimumab; nA = not available; Nivo = nivolumab; Pembro = pembrolizumab; [1,2,3] Statistics based on the total number of patients with known Breslow thickness (n = 47),

ulceration status (n = 40) and LDH serum levels (n = 38). [4] Statistics based on the total number of patients with known BOR to 1L therapy with BRAF/MEKi or CPI (n = 107)
